# Cardiac Arrest Due to Anaphylactic Shock Following Vecuronium Injection

**DOI:** 10.7759/cureus.40931

**Published:** 2023-06-25

**Authors:** Snehasis Das, Sagar Prakash, Julia Sunil, Oseen Shaikh, Gopal Balasubramanian

**Affiliations:** 1 Surgery, Jawaharlal Institute of Postgraduate Medical Education and Research, Puducherry, IND

**Keywords:** skin test, anesthesia, allergy, vecuronium, anaphylaxis

## Abstract

Anaphylaxis under anesthesia is a rare but potentially severe disease. Although anaphylaxis is rare, it can be lethal if not diagnosed and treated appropriately. We present the case of a 43-year-old male with no prior allergy history who experienced a severe anaphylactic reaction that resulted in cardiac arrest after the intravenous injection of vecuronium. His surgery was postponed, and the patient required intensive care with ventilator support and other supportive measures. Post-reaction dermal sensitivity tests revealed a clear allergic reaction to vecuronium which confirmed the diagnosis retrospectively. Eventually, the patient made a full recovery and was rescheduled for surgery at a later date.

## Introduction

Almost 60% of anesthesia-induced allergy cases are due to neuromuscular blocking drugs (NMBD) [1]. All NMBD are allergenic due to two or more haptenic determinants in their structure and the quaternary ammonium ion [2]. Most patients are unaware that they have allergies to NMBD drugs, and the allergy is often detected after the anesthesia has been induced. These allergic reactions can be mild or severe episodes, such as anaphylaxis. Skin testing helps establish a non-sensitive NMBD for general anesthesia in patients who have had an allergic reaction to an NMBD combined with a mast cell tryptase test. Determining the causal agent is the most vital step in preventing a recurrence, usually a retrospective diagnosis. Herein, we present a case history of anaphylaxis to vecuronium, a relatively uncommon sentinel occurrence. In addition, we discuss the preventative measures and management strategies for treating an anaphylactic response to vecuronium. 

## Case presentation

A 43-year-old male was diagnosed with a right inguinal hernia and was planned for total extraperitoneal repair for the right inguinal hernia. The patient had a body mass index (BMI) of 22.8 kg/m^2^. He had no underlying medical issues or allergies to drugs or food, and his family had no allergy history. No abnormalities were found during the preoperative physical examination, hematological test, imaging studies, or electrocardiography (ECG). The patient was connected to the standard key monitors showing ECG, blood pressure (BP), oxygen saturation (sPO2), and end-tidal carbon dioxide (EtCO2). He received intravenous doses of glycopyrrolate 0.25 mg, ondansetron 4 mg, and fentanyl 50 mg. Injection of thiopentone 300 mg intravenous was used to induce anesthesia. The patient was intubated with a 7.5 mm cuffed endotracheal tube (ETT) after injecting vecuronium 7 mg intravenously. To maintain anesthesia, oxygen, nitrous oxide, and isoflurane were employed. The patient's saturation dropped from 99% to 35%, he developed bradycardia to 16 beats/min, and his blood pressure could not be recorded. EtCO2 was 10 mm of Hg and was further declining. He had developed puffiness in his face. 

He started developing facial puffiness, generalized erythema, whole-body urticaria, and wheezing on auscultation. He then had an on-table cardiac arrest. The table was quickly leveled, and cardiopulmonary resuscitation (CPR) was started. He was given 200 mg of hydrocortisone intravenously and was ventilated with 100% oxygen. We gave 1 mL adrenaline 1:1000 intravenously three times. A fast infusion of one-liter crystalloid was given. His blood pressure was raised to 100/60 mm of Hg, his heart rate improved to 68 beats per minute, and his EtCO2 level was reduced to 30 mm of Hg. His blood pressure and pulse rate were fluctuating. Adrenaline injections began at 0.1 mg/kg/hr, followed by dopamine injections at 10 mg/kg/hr. His operation was postponed, and he was placed on a pressure-synchronized intermittent mandatory ventilation (P-SIMV) with a fraction of inspired oxygen (FiO2) of 0.5 (50%), 15 cm of water pressure support, and positive end-expiratory pressure (PEEP) of 5 cm of water. Over the next 24 hours, infusions were gradually tapered. He was hemodynamically stable and was removed from ventilator support the next day.

**Figure 1:**
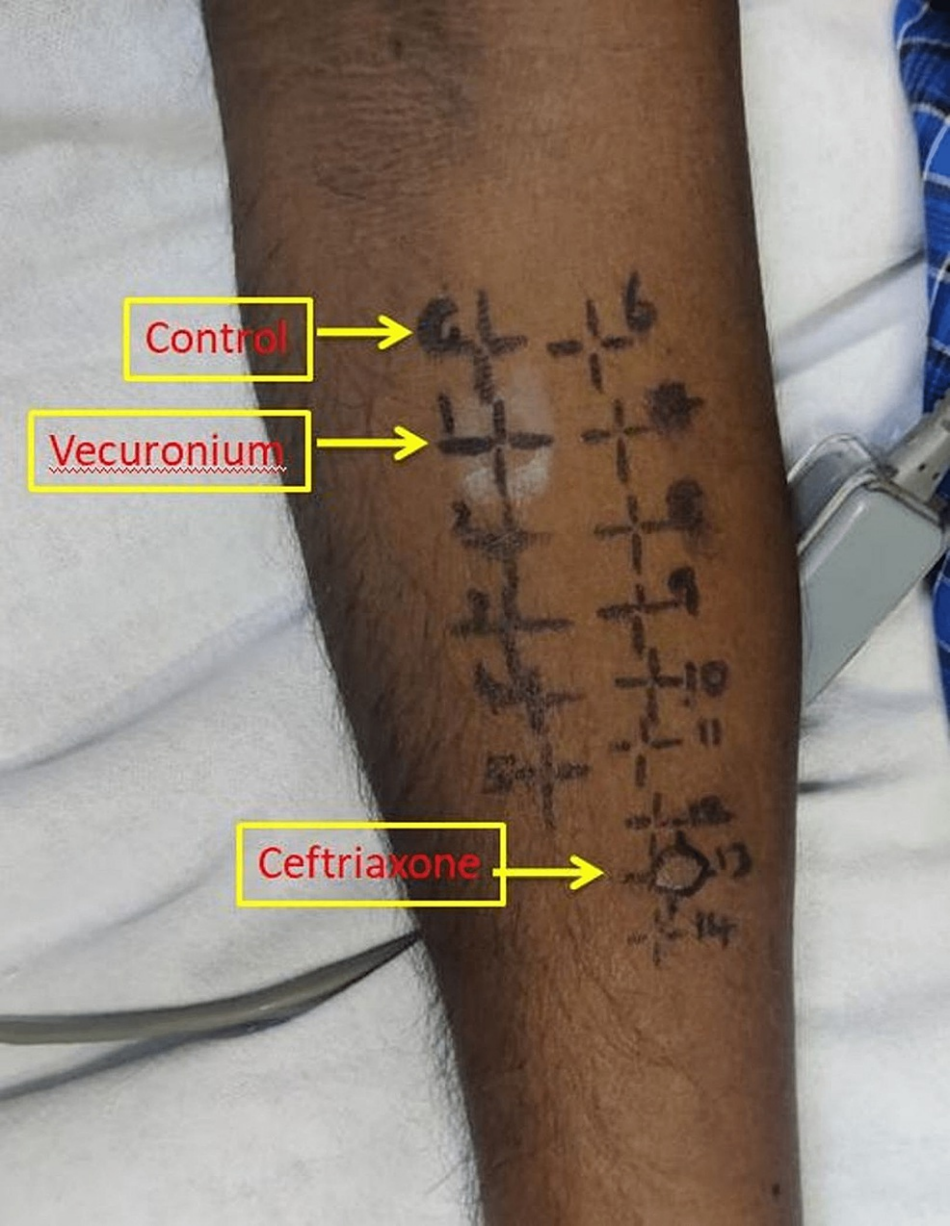
Image showing skin test for multiple drugs used during anesthesia, of which control is marked as 'C'. The patient had a strongly positive reaction to vecuronium (marked as '1') and a weakly positive reaction to ceftriaxone (marked as '13').

After stability, an intradermal skin test for the supplied medications was performed. Histamine and saline were used as positive control and negative control, respectively. These were diluted from the stock solution at 1:10, 1:100, and 1:1000 ratios. Hypodermic needles were used for the skin test. 0.03 ml of each solution was used for the test. Any wheals produced were measured 20 minutes after the injection. The injection papule's mean diameter was measured. The finding is considered significant when the diameter of the wheal produced by the tested drug is more than that of the positive control solution. This is because an intradermal skin test requires a bleb twice the size of the initial injection to be considered significant. The test was stopped if the reaction occurred at the lowest concentration level. Of the medications used to produce anesthesia on the day of the surgery, vecuronium showed the response in a 1:100 dilution. The vecuronium preparation was a 4 mg vial with no additions. Additionally, he tested positive for ceftriaxone and was labeled allergic to the medications mentioned (Figure 1).

## Discussion

NMBD causes around 50% to 60% of anaphylactic reactions under anesthesia. According to the Boston Collaborative Drug Surveillance Survey, the incidence ranges from 1:980 to 1:20,000 [2]. One episode of perioperative anaphylaxis is predicted to occur in every 10,000 to 20,000 operations worldwide [3]. The most widely used medicines that cause anaphylaxis (about 20 to 40 fatalities per year) are NMBD [4].

All NMBD are allergenic due to two or more haptenic determinants in their structure and the quaternary ammonium ion [2]. NMBD hypersensitivity reactions can be immunoglobulin E (IgE) mediated or non-IgE-mediated. The quaternary ammonium structure observed in all neuromuscular blocking medications causes cross-sensitivity between NMBD [2,5]. Anaphylaxis to succinylcholine and rocuronium is more prevalent than atracurium and vecuronium [6]. There is a high incidence of cross-sensitivity between NMBD and the severity of allergic reactions. Hence, it is recommended that NMBD be avoided in future anesthesia and that regional or local anesthetics be utilized wherever possible.

Mast cell tryptase (MCT) is enzyme-activated mast cells produce. Its concentration in serum is detectable one hour to six hours after anaphylaxis, peaking around the second hour. A beta subunit of MCT level of more than 3 ng/mL and a total MCT to beta subunit ratio of less than 10 are susceptible indications of an allergic response during anesthesia [7]. On the other hand, intradermal testing is the most helpful test for detecting the causal agent [8]. Reddy et al. confirmed that allergic reactions have higher tryptase levels and are more severe than non-allergic occurrences [9]. In our patient, we did not check MCT levels, as this test was not available in our institute.

To reduce the likelihood of perioperative anaphylaxis, no predictive tests can identify at-risk patients before intervention or reaction. As a result, secondary prevention is the most effective method [10]. Reddy et al. could not provide exact information on the risk associated with vecuronium usage due to the small number of exposures [9]. Sugammadex has been shown in large epidemiologic studies to have a lower incidence of anaphylaxis than rocuronium, and its adverse effects may be effectively reversed [11,12]. Our patient had no history of allergy to any substance or drug.

A single steroid and antihistamine dose cannot prevent an IgE-mediated hypersensitivity reaction. Antihistaminic drugs are frequently used as a prophylaxis against the reactions produced by non-IgE-mediated histamine release [13]. It was also avoided by avoiding histamine-releasing drugs and delivering medications slowly and separately. Our patient also received steroid and antihistaminic doses when the patient was diagnosed with anaphylaxis.

Precautions would involve setting up two large bore intravenous lines and preloading them with 500 ml of crystalloid solution before induction. Before induction, the patient was reoxygenated and vasodilated to offset any allergic vasodilation. According to Bouaziz and Laxenaire, if the last patch test was conducted in the past two or three years, the prior test should be repeated before anesthesia to prevent developing new sensitivities [14]. As the first line of treatment for anaphylaxis, the Japanese Society of Allergology advises intramuscular adrenaline injection [15]. Although most countries' perioperative guidelines recommend intravenous administration, there is no consensus on the intravenous dose [2]. Our patient had received intravenous adrenaline injections, as most institutions follow.

## Conclusions

It is necessary to give appropriate treatment for anaphylaxis, but it is far more important to limit exposure to its triggers. It is challenging to diagnose anaphylactic reactions intraoperatively due to the various types of drugs administered simultaneously during anesthetic induction. As a result, it is vital to obtain an early diagnosis and provide appropriate treatment when it occurs. As a result, in such cases, a high degree of suspicion and readiness to respond to anaphylaxis are essential. Despite its rarity, all physicians should be aware of vecuronium as a possible cause of anaphylaxis.

## References

[1] Gandhi R, Sharma B, Sood J, Sehgal R, Chugh P (2017). Anaphylaxis during anaesthesia: Indian scenario. Indian J Anaesth.

[2] Mertes PM, Malinovsky JM, Jouffroy L, Aberer W, Terreehorst I, Brockow K, Demoly P (2011). Reducing the risk of anaphylaxis during anesthesia: 2011 updated guidelines for clinical practice. J Investig Allergol Clin Immunol.

[3] Manandhar S (2018). Intraoperative anaphylaxis. J Patan Acad Health Sci.

[4] Takahashi K, Tanaka S, Watanabe M, Yamakage M (2019). Rocuronium-induced anaphylaxis: a case report. JA Clin Rep.

[5] Pichler WJ (2019). Immune pathomechanism and classification of drug hypersensitivity. Allergy.

[6] Reddy JI, Cooke PJ, van Schalkwyk JM, Hannam JA, Fitzharris P, Mitchell SJ (2015). Anaphylaxis is more common with rocuronium and succinylcholine than with atracurium. Anesthesiology.

[7] Valent P, Akin C, Arock M (2012). Definitions, criteria and global classification of mast cell disorders with special reference to mast cell activation syndromes: a consensus proposal. Int Arch Allergy Immunol.

[8] Michavila Gomez AV, Belver Gonzalez MT, Alvarez NC, Giner Muñoz MT, Hernando Sastre V, Porto Arceo JA, Induráin BV (2015). Perioperative anaphylactic reactions: review and procedure protocol in paediatrics. Allergol Immunopathol (Madr).

[9] Mertes PM, Volcheck GW (2015). Anaphylaxis to neuromuscular-blocking drugs: all neuromuscular-blocking drugs are not the same. Anesthesiology.

[10] Gurrieri C, Weingarten TN, Martin DP, Babovic N, Narr BJ, Sprung J, Volcheck GW (2011). Allergic reactions during anesthesia at a large United States referral center. Anesth Analg.

[11] Reitter M, Petitpain N, Latarche C (2014). Fatal anaphylaxis with neuromuscular blocking agents: a risk factor and management analysis. Allergy.

[12] Laake JH, Røttingen JA (2001). Rocuronium and anaphylaxis--a statistical challenge. Acta Anaesthesiol Scand.

[13] Kroigaard M, Garvey LH, Gillberg L (2007). Scandinavian Clinical Practice Guidelines on the diagnosis, management and follow-up of anaphylaxis during anaesthesia. Acta Anaesthesiol Scand.

[14] Agrawal N, Gogia AR, Dayal M (2017). Dilemmas in anesthetic management of a patient with history of anaphylaxis to vecuronium. Anesth Essays Res.

[15] Cardona V, Ansotegui IJ, Ebisawa M (2020). World Allergy Organization anaphylaxis guidance 2020. World Allergy Organ J.

